# Assessing Program Coverage of Two Approaches to Distributing a Complementary Feeding Supplement to Infants and Young Children in Ghana

**DOI:** 10.1371/journal.pone.0162462

**Published:** 2016-10-18

**Authors:** Grant J. Aaron, Nicholas Strutt, Nathaniel Amoh Boateng, Ernest Guevarra, Katja Siling, Alison Norris, Shibani Ghosh, Mercy Nyamikeh, Antoine Attiogbe, Richard Burns, Esi Foriwa, Yasuhiko Toride, Satoshi Kitamura, Kwaku Tano-Debrah, Daniel Sarpong, Mark Myatt

**Affiliations:** 1 Global Alliance for Improved Nutrition (GAIN), Geneva, Switzerland; 2 International Nutrition Foundation (INF), Boston, Massachusetts, United States of America; 3 University of Ghana (UG), Accra, Ghana; 4 Valid International, Oxford, England, United Kingdom; 5 Friedman School of Nutrition Science and Policy, Tufts University, Boston, Massachusetts, United States of America; 6 CARE International in Ghana, Accra, Ghana; 7 Exp Social Marketing, Accra, Ghana; 8 Ghana Health Services, Accra, Ghana; 9 Ajinomoto Co., Inc, Tokyo, Japan; 10 Brixton Health, Llawryglyn, Wales, United Kingdom; Universidade de Sao Paulo, BRAZIL

## Abstract

The work reported here assesses the coverage achieved by two sales-based approaches to distributing a complementary food supplement (KOKO Plus^™^) to infants and young children in Ghana. Delivery Model 1 was conducted in the Northern Region of Ghana and used a mixture of health extension workers (delivering behavior change communications and demand creation activities at primary healthcare centers and in the community) and petty traders recruited from among beneficiaries of a local microfinance initiative (responsible for the sale of the complementary food supplement at market stalls and house to house). Delivery Model 2 was conducted in the Eastern Region of Ghana and used a market-based approach, with the product being sold through micro-retail routes (i.e., small shops and roadside stalls) in three districts supported by behavior change communications and demand creation activities led by a local social marketing company. Both delivery models were implemented sub-nationally as 1-year pilot programs, with the aim of informing the design of a scaled-up program. A series of cross-sectional coverage surveys was implemented in each program area. Results from these surveys show that Delivery Model 1 was successful in achieving and sustaining high (i.e., 86%) effective coverage (i.e., the child had been given the product at least once in the previous 7 days) during implementation. Effective coverage fell to 62% within 3 months of the behavior change communications and demand creation activities stopping. Delivery Model 2 was successful in raising awareness of the product (i.e., 90% message coverage), but effective coverage was low (i.e., 9.4%). Future programming efforts should use the health extension / microfinance / petty trader approach in rural settings and consider adapting this approach for use in urban and peri-urban settings. Ongoing behavior change communications and demand creation activities is likely to be essential to the continued success of such programming.

## Introduction

Age-appropriate infant and young child feeding (IYCF) practices are essential to ensuring optimal child growth and development [1, 2]. For the first 6 months of life, exclusive breastfeeding is considered ideal to meet nutritional needs [1]. Later, during the complementary feeding period, children should receive safe and nutritionally adequate “complementary” foods alongside breastfeeding until 2 years of age or older [1].

Many lower-income countries, including Ghana, have made steady progress toward meeting exclusive breastfeeding goals [3]. Less has been achieved with regard to complementary feeding [3]. Recent national estimates for Ghana show that 71% of infants in the first 6 months of life are predominantly breastfed, but only 31% of children between 6 and 23 months of age are receiving optimal age-appropriate IYCF practices [4, 5] The complementary feeding period is often associated with growth faltering [6]. The prevalence of young children in Ghana classified as being stunted (i.e., having a length-for-age z-score more than two z-scores below the reference median [7, 8]) increases from about 15% at 6 months of age to more than 35% at 12 months of age [4]. Stunting has a multifactorial etiology [6], and poor complementary feeding practices are considered a major contributing factor [6]. Evidence regarding effective at-scale programming that can improve complementary feeding practices remains limited [3]. A technical meeting convened in 2008 by the World Health Organization (WHO) and the United Nations Children’s Fund (UNICEF) to strengthen actions related to complementary feeding practices concluded that there were not enough examples of well-documented large-scale programs that had been successful in improving complementary feeding practices to recommend specific program designs [3].

To improve nutrition and health outcomes during the complementary feeding period, there is a need to address issues related to availability, access, and consumption of nutrient-rich complementary foods, as well as to improve overall feeding practices in terms of meal frequency and dietary diversity [1, 3]. The work reported here is part of a broader public-private partnership (PPP) established in Ghana to address these issues, and focuses on assessing two sales-based approaches to distributing a complementary food supplement (KOKO Plus^™^) to infants and young children during the complementary feeding period [9]. Details on the project partnership structure, product development, formulation, acceptability testing, and delivery models are described elsewhere [9]. The supplement was designed for point-of-use (home fortification) as a micronutrient powder added to children’s food. The product also includes additional macronutrients, lysine, and flavorings [9]. A community-based efficacy trial evaluating the impact of use of the product on biochemical markers and linear growth was continued after the completion of the work reported here. Results for the two delivery models are presented together in this report. It should be noted that it was not an explicit objective of the research reported here to evaluate which delivery model performed better than the other. Each approach was designed to reach different populations (Delivery Model 1 for the rural poor and Delivery Model 2 for more affluent and more populous urban and peri-urban populations), with the aim of developing programming models suited for use as context-specific components of a scaled-up program. Both programs were implemented sub-nationally as 1-year pilots [9].

Delivery Model 1 was conducted in the Northern Region of Ghana and used a mixture of health extension workers (delivering behavior change communications [BCC] and demand creation activities at primary healthcare centers and in the community) and petty traders recruited from beneficiaries of a local microfinance initiative (responsible for the sale of the complementary food supplement at market stalls and house to house). Delivery Model 2 was conducted in three neighboring districts in eastern Ghana and used a purely market-based approach, with the product being sold through micro-retail routes (i.e., small shops and roadside stalls), supported by BCC and demand creation activities led by a local social marketing company.

BCC and demand creation activities included generic IYCF promotion by Ghana Health Services (both models), cookery demonstrations and tastings (both models), billboards and posters (both models), house-to-house sales (specifically in model 1 as door-to-door hawkers were not targeted by model 2), songs-based and street-theatre based messaging (model 1 only), nutrition and health education (model 1 only), distribution of free samples to beneficiaries at health facilities and at points of sale (model 2 only), distribution of free samples to potential sales outlets (model 2 only), radio news and talk-shows (model 2 only), product placement in radio soap operas (model 2 only), community discussions with consumer groups (model 2 only), and mobile public address system (model 2 only).

The main objective of the research was to assess the effectiveness of the two delivery models with regard to program coverage.

## Methods

### Delivery Model 1

#### Program setting and survey population

Program implementation for Delivery Model 1 was carried out by a nongovernmental organization (NGO), CARE International in Ghana, in 13 neighboring rural communities in the East Mamprusi District of Ghana’s Northern Region. This region is much drier than southern areas of the country; the region experiences 3 months of rainfall annually between June and September, with a dry season extending from November to April [10]. Subsistence farming is the main source of income and is limited to staple grains and legumes [10]. Because of climate and distance from commercial centers, the Northern Region is one of the poorest and most food insecure regions of Ghana [10]. The target consumer age group for program delivery was children aged 6 months and over, with particular emphasis on the complementary feeding period from 6 to 24 months. The survey population consisted of children aged between 6 and 24 months and their principal caregivers (defined as the person who provides most care for the child and gives the child most meals on most days).

#### Survey design and sample size

A pilot survey was implemented before the start of the program to train survey staff and to test questionnaires and indicators (results not reported here). Three coverage surveys were implemented during the program delivery period. Survey rounds one and two were implemented at 3 and 10 months into the program, respectively. Survey round three was implemented 14 months after the start of the program. This was 3 months after BCC and demand creation activities had ceased. Survey samples were independent of each other. The surveys employed a two-stage sampling procedure. The first stage consisted of all 13 intervention communities as primary sampling units (PSUs). The second stage consisted of households sampled from each PSU using the quarter (QTR) method (i.e., division of a community into four areas of approximately equal population size) and a random walk (EPI3) sampling method in each quarter [11]. The EPI3 method selects the first household in a quarter to be sampled using the EPI strategy, with subsequent households selected by choosing a random direction and selecting the third nearest house in that direction [11]. This method has been shown to yield results comparable to simple random samples and to be better than the unmodified EPI sampling strategy when a wide range of indicators is being assessed [11].

Sample sizes were calculated for estimating a proportion with a finite population correction [12]. Assuming an expected coverage of 50%, a desired precision (i.e., half-width of the 95% confidence interval) of ± 8%, a maximum expected survey design effect of 2.0, and a population of *N* ≈ 12,000, the sample size required was calculated to be *n* = 300 households per survey. This was increased to *n* = 312 households per survey (giving *n* = 24 households from each village) to simplify partitioning of the within-community sample into quarters as required by the QTR sampling method.

### Delivery Model 2

#### Program setting and survey population

Delivery Model 2 was implemented by the local not-for-profit arm of a pan-African social marketing company (Exp Social Marketing—ESM) in three neighboring districts (Nsawam, Suhum, and Asamankese) in the Eastern Region of Ghana. These districts were selected because of their large urban and peri-urban populations, their proximity to Accra, and to avoid interfering with other regions in Ghana where known nutritional trials were ongoing. Income levels vary across the three districts depending on the degree of urbanization. Subsistence farming and petty trade dominate in rural areas. Commerce, manufacturing, building, and service sector activities dominate in the urban and peri-urban areas [10]. The Eastern Region benefits from substantial annual rainfall and a second growing season, supporting a more productive and varied agriculture than is possible in the Northern Region [10]. Poverty and food insecurity, while present, are both less severe and less prevalent than in the Northern Region [10].

The target consumer age group for the program was the same as in Delivery Model 1 (i.e., children aged between 6 and 24 months). The coverage surveys reported here sampled children aged between 0 and 24 months. The rationale for including younger children was to pilot a simple structured IYCF indicator set for use elsewhere. Data for children aged under 6 months are not presented here.

#### Survey design and sample size

Two coverage surveys were implemented during the program delivery period. Survey round one was implemented at 2 months into the program, and round two at 11 months into the program. Survey samples were independent of each other. The surveys were designed to be spatially representative, that is, the sample was distributed evenly across the survey area, using a spatial sample design that selected communities located closest to the centroids of a hexagonal grid laid over the survey area. The resulting sample is a triangular irregular network [13, 14]. A variable intensity sampling design was used [13]. In rural areas, the sample density was such that no person lived more than about 8 kilometers from a sampling point. Sampling density increased with increasing population density. Each survey used a sample of *n* = 18 caregiver-child pairs from *m* = 58 PSUs (villages or city blocks). The within-community sample in villages used systematic sampling of dwellings in the villages (or parts of villages) organized as a ribbon (or ribbons) of dwellings, and a random walk EPI3 sampling strategy in villages (or parts of the villages) organized as clusters of dwellings. Sampling in urban communities used systematic household sampling with a sampling interval calculated in the field. This sample design provides *implicit stratification*, selecting a sample that is distributed across both the entire survey area and within sampled communities [15]. This type of sample tends to spread the sample among important subgroups of the population, (e.g., rural, urban, and peri-urban; different administrative areas; ethnic / religious subpopulations; and various socioeconomic groups) and often improves the precision of estimates made from survey data [15, 16].

### Ethical clearance and survey administration procedures

Ethical clearance to conduct the coverage surveys for both delivery models was obtained from the Ghana Health Services Ethical Review Committee (protocol ID number GHS-ERC-05092012). Oral consent to participate was obtained from the child’s principal caregiver on the basis that participation in the survey was voluntary. Written consent was not sought due to concerns regarding the adult female literacy rate in Ghana. Consent was recorded in survey supervisors’ logbooks. The Ghana Health Services Ethical Review Committee approved this consent process. Trained interviewers under the supervision of experienced field supervisors collected data. For Delivery Model 1, data were collected using mobile devices (Open Data Kit version 1.3) with pre-coded logical responses to ensure data quality. For Delivery Model 2, data were collected using paper forms, with data entry and interactive checking (for consistency, ranges, and legal values during data entry) and batch checking (double-entry and validation, as well as a batch application for consistency, range, and legal value checks) performed using EpiData (version 3.1) [17].

### Survey instrument

Data were collected on demographics and socioeconomic status; education levels within the household; housing conditions; recent infant and child mortality; water, sanitation, and hygiene (WASH) practices; food security; child health; IYCF practices; maternal dietary diversity; coverage of fortified staples; product coverage; and maternal and child anthropometry.

The same survey instrument was used in both sets of delivery model assessments. All survey modules (i.e., question and indicator sets) were taken from validated guidelines with language, wording, and layout finalized through pilot testing in the field. All case-definitions (e.g., for maternal and child undernutrition, hunger, poor sanitation, and suboptimal IYCF practices) adhered to internationally recognized standards. Product coverage question sets and indicators were adapted from those used in semi-quantitative evaluation of access and coverage (SQUEAC) and simplified lot quality assurance evaluation of access and coverage (SLEAC) coverage assessments [18].

### Indicators of risk and need

Three key indicators of risk (or need) were used to investigate the targeting efficiency of the two delivery models. These were poverty, poor maternal dietary diversity, and suboptimal IYCF practices.

**Fig 1:**
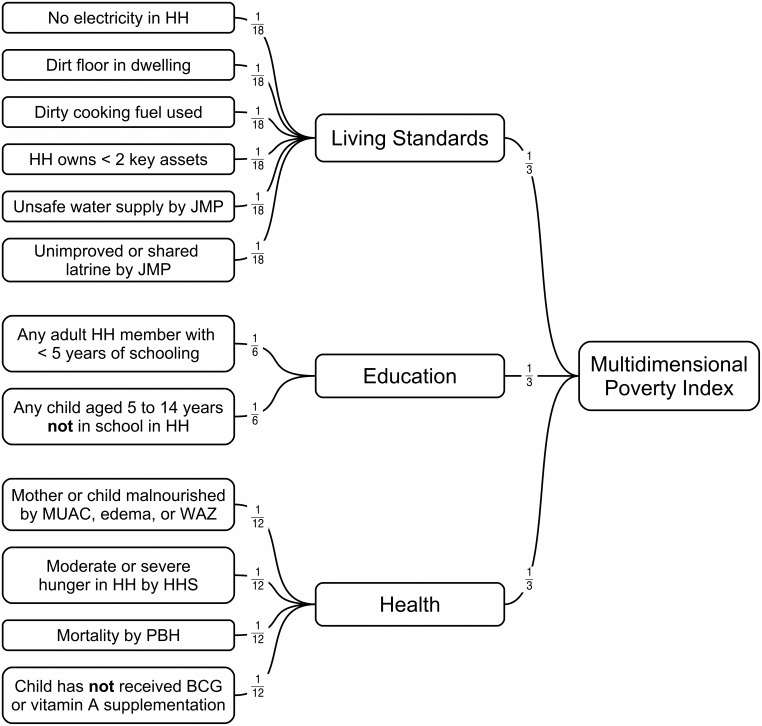
Component indicators and weightings used to calculate the MPI. HH = Household; HHS = Household Hunger Score; JMP = WHO/UNICEF Joint Monitoring Program for Water Supply and Sanitation; MUAC = Mid-upper arm circumference; PBH = Previous birth history; BCG = Bacillus Calmette–Guérin vaccine; WAZ = Weight-for-age z-score (WHO Growth Standards); Edema = the presence of bilateral pitting edema.

Poverty was assessed using an adapted multidimensional poverty index (MPI) [19]. Adaptations followed published guidelines. The MPI score is constructed as a weighted sum of indicators in three dimensions (health, education, and living standards) and ranges between 0 and 1. Fig 1 shows the component indicators and weightings used to calculate the MPI score used in the assessments reported here. A household was classified as being in poverty if the MPI score was greater than or equal to one third.

Maternal dietary diversity was assessed using the Women’s Dietary Diversity Score (WDDS), which is a count of food groups (from a list of nine food groups) consumed in the previous 24 hours [20]. Poor maternal dietary diversity was defined as having a WDDS below the sample median WDDS.

Suboptimal IYCF practices were assessed using an Infant and Child Feeding Index (ICFI) [21, 22]. All children aged between 6 and 24 months received an ICFI score between 0 and 6. The ICFI score is a measure of age-appropriate child feeding practices using age-appropriate scoring for breastfeeding, dietary diversity, and meal frequency (Table 1). Children with a total score less than 6 were classified as having suboptimal IYCF practices.

**Table 1:** ICFI Scoring Scheme for Age-Appropriate IYCF Practices. [22]

	Age group (months)					
	6–8		9–11		12–24	
IYCF Practice	Value	Score	Value	Score	Value	Score
Breastfed (previous 24 hours)	Yes	+2	Yes	+2	Yes	+1
Food groups (previous 24 hours)	1	+1	1 or 2	+1	2 or 3	+1
	≥ 2	+2	≥ 3	+2	≥ 4	+2
Meal frequency (previous 24 hours)	1	+1	1 or 2	+1	2	+1
	≥ 2	+2	≥ 3	+2	3	+2
					≥ 4	+3

Children with a total score less than 6 are classified as having suboptimal IYCF practices.

### Indicators of coverage

Three measures of coverage were assessed following the model of Tanahashi [23]: “message coverage” (i.e., has the caregiver ever heard of the product?), “contact coverage” (i.e., has the child ever been fed the product?), and “effective coverage” (i.e., has the child been fed the product at least once in the previous 7 days?).

**Fig 2:**
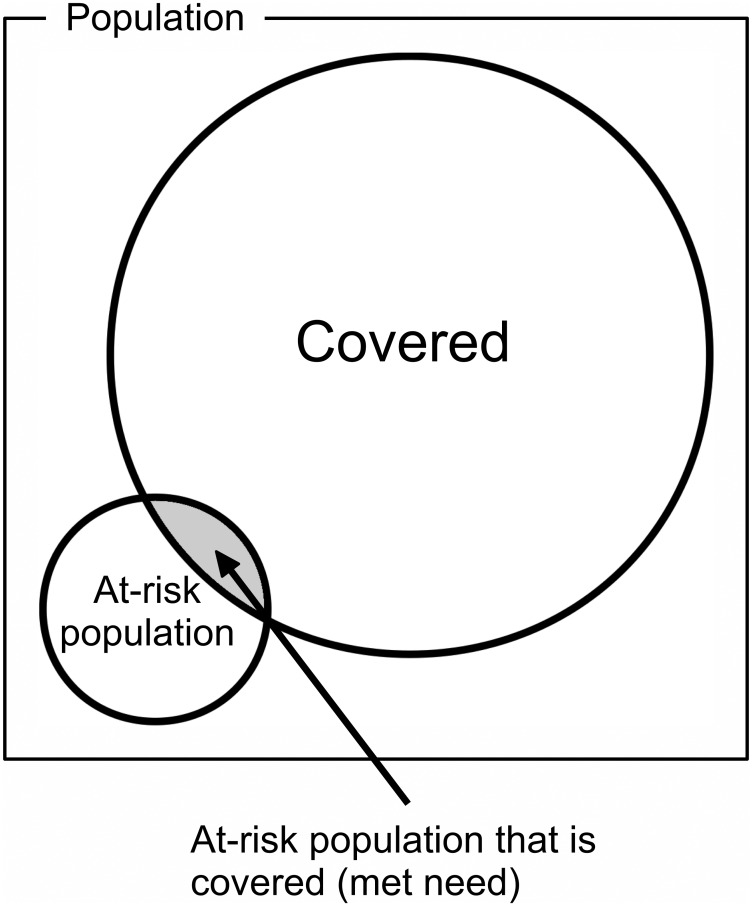
MN Is the Proportion of Children Defined as At-Risk Who Are Covered.

Three summary statistics were estimated for each of the three coverage measures:

- “Raw coverage” (RC) is the proportion of all children who are covered. This is a measure of overall program performance.
- “Met need” (MN) is the proportion of children defined as at-risk who are covered (Fig 2). This is a measure of how well a delivery model addresses need.
- The “Coverage ratio” (CR) is the ratio of the coverage in children defined as at-risk by a specific indicator (e.g. poor IYCF) to the coverage in children not defined as at-risk by the same indictor (e.g. good IYCF). CR is a measure of how well a program targets need. The CR ranges between 0 and positive infinity. CR values below 1 indicate poor targeting (i.e., coverage is higher in the not-at-risk population than in the at-risk population). CR values above 1 indicate good targeting (i.e., coverage is higher in the at-risk population than in the not-at-risk population). A CR of 1 indicates an absence of targeting (i.e., coverage is similar in the at-risk and not-at-risk populations).

**Fig 3:**
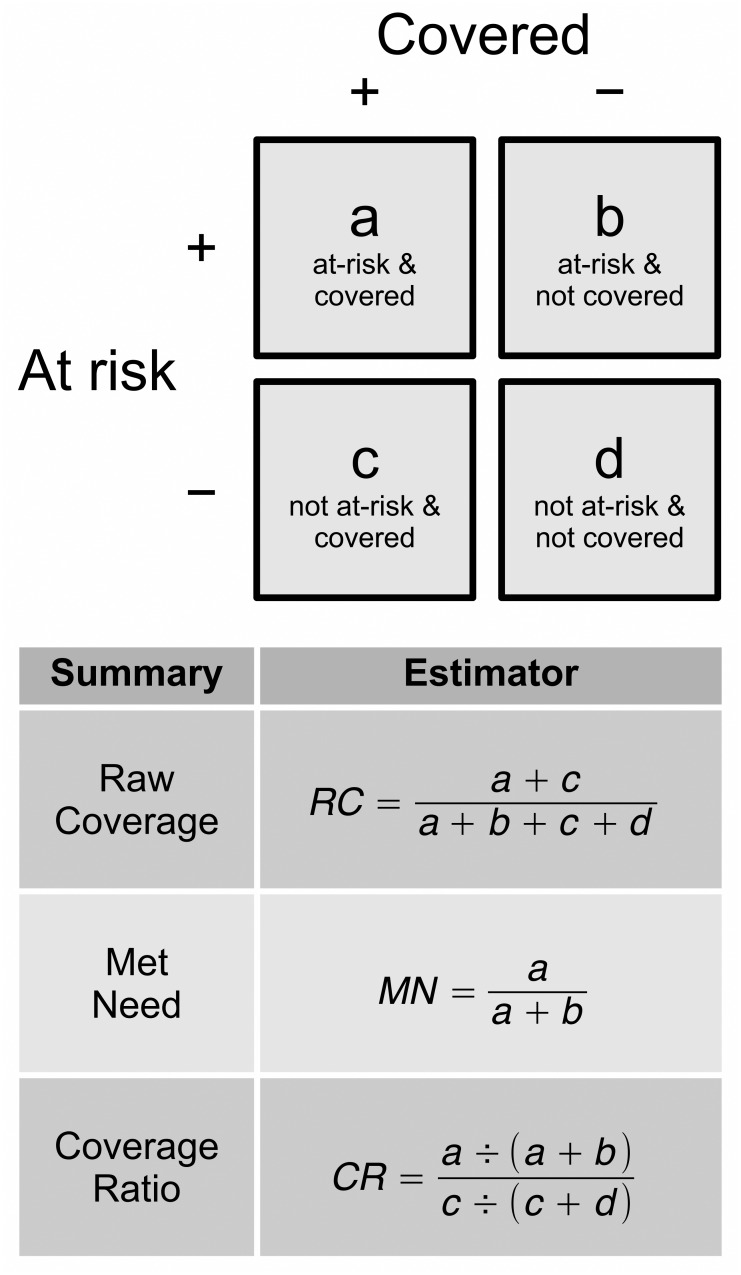
Two-by-Two Table Showing the Definitions of RC, MN, and CR.

A program may be classified as well functioning based on observing a high RC or efficient targeting of need (i.e., high MN with a CR above 1). Fig 3 shows how the three summary statistics are calculated from a two-by-two table.

### Data analyses

Data were analyzed using the R language for data analysis and graphics (version 3.1.2) and the R-AnalyticFlow scientific workflow system (version 3.01). A blocked weighted bootstrap estimation technique was used [24]. Bootstrap replicates consisted of a set of within-PSU survey samples that were sampled with replacement and with a probability proportion to PSU population size using a *roulette wheel* (also known as *stochastic sampling with replacement*) algorithm [25]. For each bootstrap replicate, a total of *m* PSUs were sampled with replacement (where *m* is the number of PSUs in the survey sample). Observations within selected PSUs were also sampled with replacement with the same within-PSU sample size that was achieved in the survey. A total of *r* = 400 bootstrap replicates were used. The required summary statistic was calculated from each replicate. The resulting estimate consisted of the 2.5th (lower 95% confidence limit), 50th (point estimate), and 97.5th (upper 95% confidence limit) percentiles of the distribution of the statistic across all replicates [26]. This procedure accounts for unequal selection probabilities in the sample design (by applying posterior weighting), as well as for any variance lost due to the clustered nature of the sample [24].

Coverage observed at each survey round was compared with the coverage observed at the previous survey round using a two-sample z-test. Individual standard errors were calculated as:

$$SE_{coverage}=\frac{UCL-LCL}{2\times 1.96}$$

where *UCL* and *LCL* are the upper and lower 95% confidence limits on the coverage proportion. The resulting standard errors were pooled:

$$SE_{pooled}=\sqrt{SE_{t}^{2}+SE_{t-1}^{2}}$$

and the test-statistic calculated as:

$$z=\frac{\left|Coverage_{t}-Coverage_{t-1}\right|}{SE_{pooled}}$$

A two sided p-value was calculated.

The design of the sample used for the assessment of coverage of Delivery Model 2 allowed for results to be mapped. RC for message, contact, and effective coverage were mapped. Interpolation between sampling points was performed using inverse distance weighting, using a global neighborhood with the weighting power that minimized errors in a twofold “holdout” cross-validation [27, 28].

## Results

### Characteristics of survey samples

Characteristics of the survey samples in Delivery Models 1 and 2 are shown in Tables 2 and 3. Survey durations ranged between 2 and 3 weeks per survey round. Preliminary results from each survey were reported back to the programs within 2 weeks of survey completion to provide feedback to guide programming efforts.

**Table 2:** Sample Description for Delivery Model 1 Assessments.

Variable	Survey round		
	Round 1	Round 2	Round 3
	(Month 3)	(Month 10)	(Month 14)
Sample size (PSUs)^a^	306 (13)	306 (13)	307 (13)
Age of caregiver^b^ in years, median (range)	30.0 (18.0, 50.0)	29.0 (17.0, 65.0)	28.0 (17.0, 46.0)
Age of child in months, median (range)	14.0 (6.0, 23.0)	16.0 (6.0, 24.0)	14.0 (6.0, 23.0)
Sex of child, % male (95% CI)	53.2% (46.2%, 59.9%)	59.4% (52.6%, 65.7%)	54.8% (46.6%, 62.5%)

^a^ Number of caregiver-child pairs surveyed (PSUs).

^b^ The caregiver was most frequently the child’s mother but, in cases of maternal absence, it may have been an older sibling, a paid caregiver/servant, or a grandparent.

**Table 3:** Sample Description for Delivery Model 2 Assessments.

Variable	Survey round	
	Round 1	Round 2
	(Month 2)	(Month 11)
Sample size (PSUs)^a^	620 (58)	663 (58)
Age of caregiver^b^ in years, median (range)	28.5 (15.0, 74.0)	28.1 (14.0, 64.0)
Age of child^c^ in months, median (range)	14.3 (6.0, 24.0)	14.5 (6.0, 24.0)
Sex of child, % male (95% CI)	52.5% (47.0%, 57.2%)	50.1% (45.0%, 54.4%)

^a^ Number of caregiver-child pairs surveyed (PSUs). The numbers reported are for children aged between 6 and 24 months. The number of children aged between 0 and 24 months sampled was 971 in Round 1 and 928 in Round 2.

^b^ The caregiver was most frequently the child’s mother but, in cases of maternal absence, it may have been an older sibling, a paid caregiver/servant, or a grandparent.

^c^ These surveys collected data for children aged between birth and 2 years. Coverage results presented in this report are for children aged between 6 and 24 months because this was the target age range for the intervention.

### Patterns of risk, program coverage, met need, and coverage ratios

**Fig 4:**
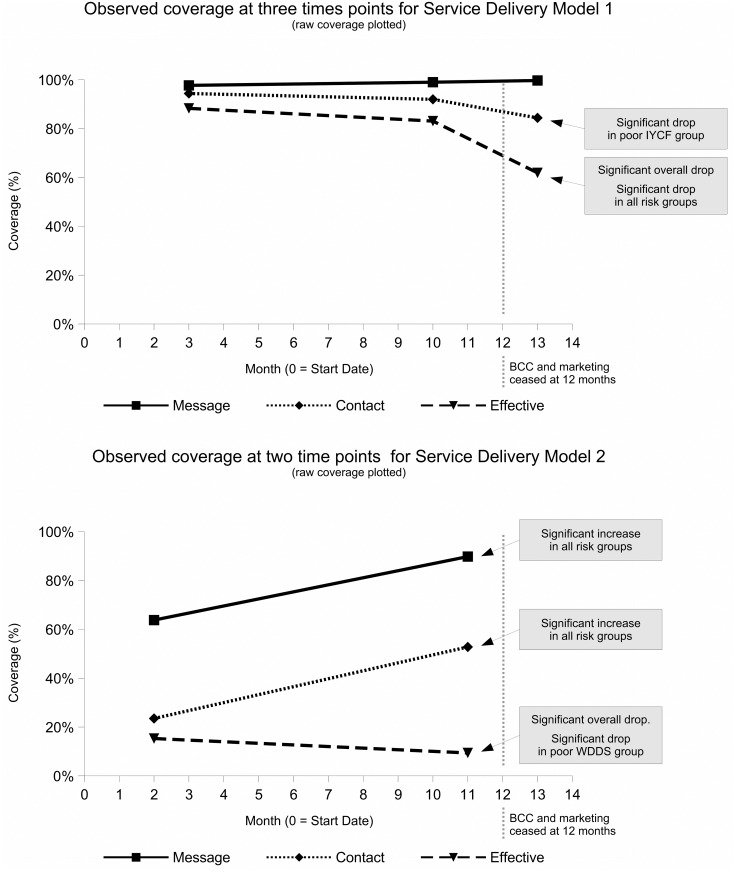
Graphs Showing Observed Coverage Measures for Both Delivery Models.

**Fig 5:**
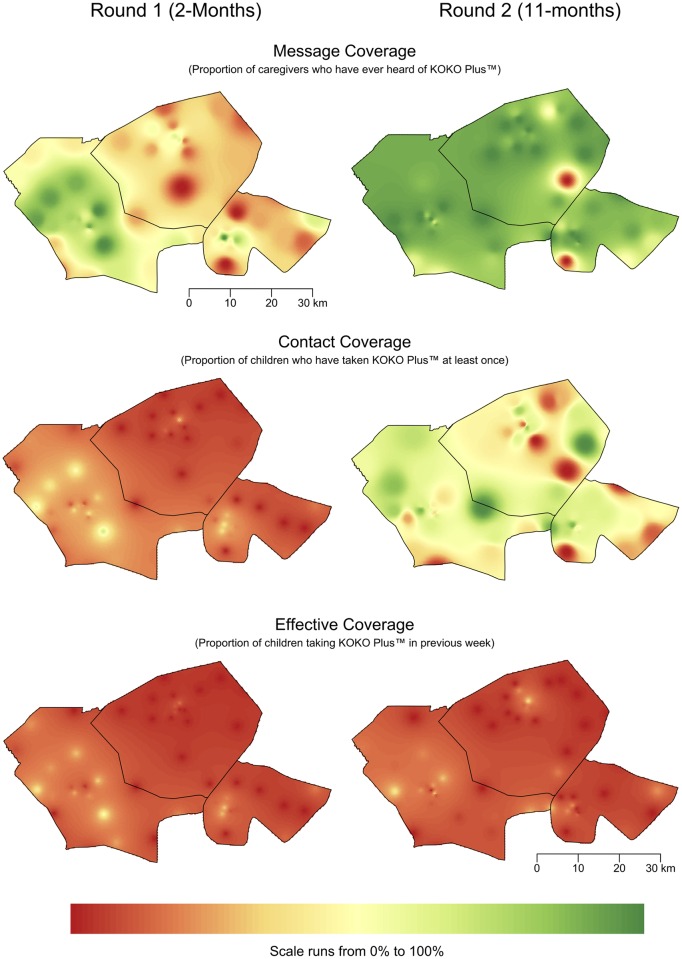
Maps of the Raw Coverage Achieved by Delivery Model 2.

Summary statistics by survey round and each measure of coverage (overall and for each at-risk group) are shown in Table 4 for Delivery model 1 and Table 5 for Delivery model 2. The results for raw coverage for both delivery models are also presented graphically in Fig 4. Maps of coverage achieved by Delivery Model 2 are shown in Fig 5. Delivery Model 1 achieved and sustained high message, contact, and effective coverage during the program implementation period with no significant differences observed between survey rounds 1 and 2 (Table 4 and Fig 4). Contact coverage dropped significantly between survey rounds 2 and 3 in the poor IYCF group (p = 0.0049). Effective coverage dropped significantly between survey rounds 2 and 3 overall (p = 0.0013), in the poverty group (p = 0.0060), in the poor WDDS group (p = 0.0090), and in the poor IYCF group (p = 0.0005). Delivery Model 2 achieved high message coverage, moderate contact coverage, and low effective coverage during the program implementation period (Table 5 and Fig 4). Message coverage and contact coverage increased significantly overall and in all risk groups between survey rounds 1 and 2 (Table 5). Effective coverage between survey rounds 1 and 2 decreased overall (p = 0.0238) and in the poor WDDS group (p = 0.0014).

**Table 4:** Risk, Met Need, and Coverage Ratios for Delivery Model 1 Assessments. ^a^

		Survey round									Notes
		Round 1			Round 2			Round 3			
		(Month 3)			(Month 10)			(Month 14)			
Coverage measure	Risk group	% at-risk^b^ (95% CI)	% MN^c^ (95% CI)	CR^d^ (95% CI)	% at-risk (95% CI)	% MN (95% CI)	CR (95% CI)	% at-risk (95% CI)	% MN (95% CI)	CR (95% CI)	
**Message**	**ALL**^e^	-	97.7 (92.9, 100.0)	-	-	99.0 (91.8, 100.0)	-	-	99.7 (98.4, 100.0)	-	
	**Poverty**^f^	74.7 (62.1, 85.3)	97.0 (92.2, 99.6)	1.00 (0.95, 1.04)	67.9 (53.6, 82.8)	99.0 (87.1, 100.0)	1.00 (0.90, 1.05)	58.3 (49.0, 67.8)	100.0 (98.6, 100.0)	1.01 (0.99, 1.05)	
	**WDDS**^g^	39.0 (28.2, 52.0)	100.0 (100.0, 100.0)	1.05 (1.01, 1.13)	44.3 (31.9, 61.5)	96.4 (86.1, 100.0)	0.99 (0.89, 1.01)	20.2 (13.5, 27.3)	96.8 (90.1, 100.0)	0.97 (0.90, 1.00)	
	**IYCF**^h^	51.6 (41.3, 61.8)	98.0 (94.0, 100.0)	1.01 (0.98, 1.08)	56.1 (45.2, 68.2)	99.0 (95.2, 100.0)	1.02 (0.99, 1.12)	60.3 (52.6, 67.0)	98.9 (97.0, 99.5)	0.98 (0.97, 1.00)	
**Contact**	**ALL**	-	94.4 (89.7, 98.1)	-	-	92.0 (82.7, 98.7)	-	-	84.4 (77.6, 89.9)	-	
	**Poverty**	74.7 (62.1, 85.3)	93.6 (89.2, 97.8)	0.98 (0.91, 1.06)	67.9 (53.6, 82.8)	89.3 (77.8, 99.4)	0.93 (0.80, 1.04)	58.3 (49.0, 67.8)	82.6 (74.4, 90.6)	0.95 (0.84, 1.10)	
	**WDDS**	39.0 (28.2, 52.0)	98.3 (94.5, 100.0)	1.06 (1.00, 1.15)	44.3 (31.9, 61.5)	85.7 (69.4, 99.1)	0.88 (0.72, 1.02)	20.2 (13.5, 27.3)	82.6 (68.5, 94.5)	0.97 (0.82, 1.11)	
	**IYCF**	51.6 (41.3, 61.8)	93.2 (88.1, 97.4)	0.97 (0.91, 1.06)	56.1 (45.2, 68.2)	95.9 (89.1, 99.1)	1.09 (0.98, 1.28)	60.3 (52.6, 67.0)	80.8 (71.5, 90.0)	0.90 (0.77, 1.04)	Significant drop between rounds 2 & 3 (*p* = 0.0049)
**Effective**	**ALL**	-	88.3 (81.1, 94.6)	-	-	83.1 (73.4, 93.1)	-	-	61.9 (53.2, 69.9)	-	Significant drop between rounds 2 & 3 (*p* = 0.0013)
	**Poverty**	74.7 (62.1, 85.3)	86.0 (78.0, 93.2)	0.90 (0.80, 1.03)	67.9 (53.6, 82.8)	85.5 (72.9, 95.8)	1.09 (0.88, 1.42)	58.3 (49.0, 67.8)	62.1 (50.2, 74.5)	1.00 (0.77, 1.27)	Significant drop between rounds 2 & 3 (*p* = 0.0060)
	**WDDS**	39.0 (28.2, 52.0)	89.9 (76.1, 98.1)	1.03 (0.87, 1.16)	44.3 (31.9, 61.5)	79.1 (59.3, 93.5)	0.91 (0.68, 1.10)	20.2 (13.5, 27.3)	47.2 (30.6, 64.1)	0.73 (0.49, 1.05)	Significant drop between rounds 2 & 3 (*p* = 0.0090)
	**IYCF**	51.6 (41.3, 61.8)	84.5 (73.1, 92.4)	0.91 (0.79, 1.06)	56.1 (45.2, 68.2)	87.1 (76.2, 94.9)	1.11 (0.97, 1.32)	60.3 (52.6, 67.0)	60.5 (49.7, 73.3)	0.97 (0.76, 1.23)	Significant drop between rounds 2 & 3 (*p* = 0.0005)

^a^ All values are percent (95% CI), unless otherwise indicated; all p-values are two-sided p-values for a two sample z-test.

^b^ At-risk is the proportion of children in the at-risk group.

^c^ MN is the estimated coverage in the at-risk group. MN is RC for the ALL risk group.

^d^ The ratio of coverage in children defined as at-risk by a specific indicator (e.g. poor IYCF) to the coverage in children not defined as at-risk by the same indictor (e.g. good IYCF).

^e^ All children aged between 6 and 24 months inclusive.

^f^ Household in poverty as classified by the MPI.

^g^ WDDS below sample median.

^h^ IYCF—suboptimal IYCF practices as classified by the Infant Child Feeding Index (ICFI).

**Table 5:** Risk, Met Need, and Coverage Ratios for Delivery Model 2 Assessments. *

		Survey round						Notes
		Round 1			Round 2			
		(Month 2)			(Month 11)			
Coverage measure	Risk group	% at-risk^a^ (95% CI)	% MN^b^ (95% CI)	CR^c^ (95% CI)	% at-risk (95% CI)	% MN (95% CI)	CR (95% CI)	
**Message**	**ALL** ^d^	-	63.8 (57.2, 71.1)	-	-	89.8 (86.6, 92.4)	-	Increase between rounds (p < 0.0001)
	**Poverty**^e^	17.6 (13.6, 21.6)	67.9 (53.7, 80.3)	1.07 (0.88, 1.25)	14.4 (10.5, 17.9)	89.2 (81.0, 96.1)	0.99 (0.90, 1.07)	Increase between rounds (p = 0.0063)
	**WDDS**^f^	45.7 (41.7, 50.1)	61.3 (52.0, 69.7)	0.92 (0.76, 1.09)	34.8 (31.1, 38.6)	88.5 (81.8, 93.0)	0.97 (0.90, 1.04)	Increase between rounds (p < 0.0001)
	**IYCF**^g^	70.4 (65.7, 74.8)	63.4 (55.5, 72.4)	0.99 (0.85, 1.18)	76.9 (72.7, 81.2)	90.8 (87.2, 93.4)	1.05 (0.98, 1.14)	Increase between rounds (p < 0.0001)
**Contact**	**ALL**	-	23.5 (19.0, 28.5)	-	-	52.8 (47.7, 58.9)	-	Increase between rounds (p < 0.0001)
	**Poverty**	17.6 (13.6, 21.6)	22.8 (14.3, 32.6)	0.99 (0.62, 1.43)	14.4 (10.5, 17.9)	45.9 (36.2, 55.6)	0.85 (0.65, 1.07)	Increase between rounds (p = 0.0007)
	**WDDS**	45.7 (41.7, 50.1)	26.7 (20.7, 33.7)	1.32 (0.96, 1.80)	34.8 (31.1, 38.6)	54.8 (45.2, 62.8)	1.04 (0.85, 1.23)	Increase between rounds (p < 0.0001)
	**IYCF**	70.4 (65.7, 74.8)	20.1 (14.6, 26.0)	0.67 (0.44, 0.99)	76.9 (72.7, 81.2)	55.6 (49.1, 60.7)	1.22 (0.96, 1.60)	Increase between rounds (p < 0.0001)
**Effective**	**ALL**	-	15.3 (11.3, 19.8)	-	-	9.4 (6.7, 12.4)	-	Decrease between rounds (p = 0.0238)
	**Poverty**	17.6 (13.6, 21.6)	19.1 (11.1, 27.7)	1.35 (0.70, 2.22)	14.4 (10.5, 17.9)	12.7 (6.7, 19.3)	1.42 (0.71, 2.67)	
	**WDDS**	45.7 (41.7, 50.1)	17.9 (12.2, 23.7)	1.38 (0.89, 2.36)	34.8 (31.1, 38.6)	6.5 (3.1, 11.1)	0.57 (0.28, 1.13)	Decrease between rounds (p = 0.0014)
	**IYCF**	70.4 (65.7, 74.8)	14.1 (10.4, 18.7)	0.81 (0.52, 1.33)	76.9 (72.7, 81.2)	9.2 (6.4, 12.5)	0.93 (0.53, 2.03)	

* All values are percent (95% CI), unless otherwise indicated; all p-values are two-sided p-values for a two sample z-test.

^a^ At-risk is the proportion of children in the at-risk group.

^b^ Met need (MN) is the estimated coverage in the at-risk group. MN is raw coverage (RC) for the ALL risk group.

^c^ The ratio of coverage in children defined as at-risk by a specific indicator (e.g. poor IYCF) to the coverage in children not defined as at-risk by the same indictor (e.g. good IYCF).

^d^ All children aged between 6 and 24 months inclusive.

^e^ Household in poverty as classified by the Multidimensional Poverty Index (MPI).

^f^ Women’s dietary diversity score (WDDS) below sample median.

^g^ IYCF—sub-optimal infant and young child feeding practices as classified by the Infant Child Feeding Index (ICFI).

## Discussion

The results presented here contribute to information gaps about the effectiveness of sales-based delivery channels for distributing home fortification products to infants and young children during the complementary feeding period.

Delivery Model 1 achieved and sustained high message, contact, and effective coverage during the program implementation period. Delivery Model 2 achieved high message coverage, moderate contact coverage, and low effective coverage.

**Fig 6:**
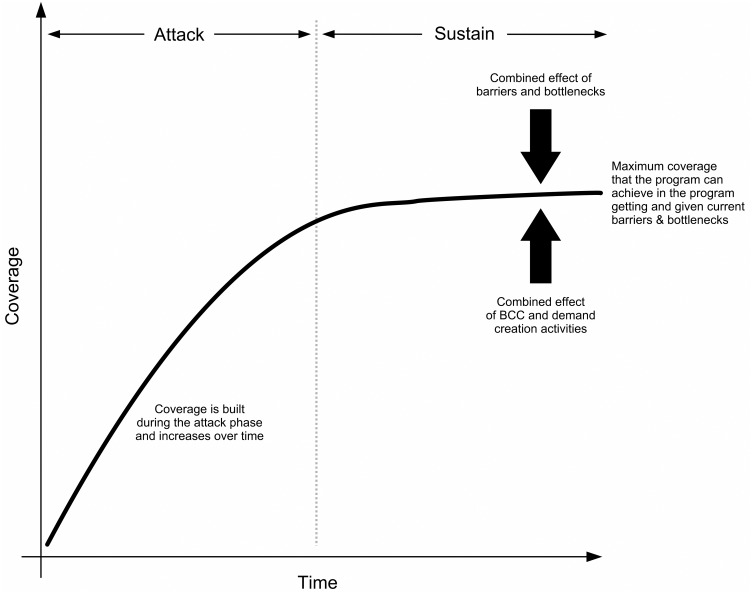
A simple Model of How Program Coverage Changes over Time.

Fig 6 shows a simple model of how program coverage changes over time [29]. Assuming a new intervention, coverage is zero at the start of the program and, providing that appropriate design decisions have been made and proper attention has been paid to BCC and demand creation, then coverage should increase until a limit (determined by barriers and bottlenecks in the program) is reached.

Key questions are “How long is the duration of this ‘attack’ phase?” and “What level of coverage can, eventually, be achieved by a given program?” These are not simple questions. The answers will vary by context and program type. In the work reported here, it is clear that Delivery Model 1 can achieve high coverage over short time periods (e.g., 88.3% effective coverage was achieved within 3 months of the start of the program) and that these high coverage levels can be sustained. It is also clear from the significant drop in effective coverage observed by the round 3 survey for Delivery Model 1 that ongoing BCC and other demand creation activities are essential to sustaining high levels of coverage. The effects of marketing activities on sales is well established in consumer market-research literature [30]. There are, however, few examples of nutrition programs operating on a sales / cost-recovery basis that have assessed long-term sustainability. In one study in the Western Region of Kenya, which assessed sales of micronutrient powders 18 months after marketing activities ended, program coverage had fallen from 64.9% to 21.9% [31].

A 1-year program implementation period may be a short time frame for conducting an assessment of a market-based approach such as was used for Delivery Model 2. Achieving 90% message coverage and 53% contact coverage within 1 year are respectable outcomes. Effective coverage at 9.4% after 11 months is, in the absence of complementary delivery modes, a very poor result. It may be that a longer assessment would have seen higher levels of effective coverage being achieved over time. The observed fall in effective coverage coincident with increases in both message and contact coverage suggests, however, that the current programming model is unable to achieve high levels of effective coverage if used as the sole delivery mode. This may, for example, have been due to the insufficient mobilization of salespersons and businesses to stock, promote, and sell the product or to a lack of focus on exploiting existing “business-to-business” supply systems and networks rather than focusing solely on small retail outlets. In both of these cases the concentration on demand rather than supply means the engagement with markets remains superficial.

Neither of the delivery models was designed to explicitly target children at risk and, unsurprisingly, neither program achieved coverage ratios significantly different from one for the risk factors assessed. This is not a strongly negative outcome, as this result shows that the program was not excluding children at risk.

It is possible that coverage was influenced by the use of health workers. It could (e.g.) be argued that the presence of health professionals conveys “warm” ideas of care and guidance, whilst a marketing approach could convey “cold” ideas of trade and profit and that this may have hindered uptake and ongoing use of the product. In model 1, KOKO Plus^™^ was delivered by petty traders recruited from beneficiaries of a local microfinance initiative supported by CARE. BCC and demand creation activities included generic IYCF promotion delivered by health extension workers (HEWs) employed by Ghana Health Services (GHS). These activities did not include the promotion of a single product. KOKO Plus^™^ was not directly promoted (i.e. not mentioned) by GHS personnel. CARE promoted the use of KOKO Plus^™^ in (e.g.) cooking demonstrations and songs-based and community theatre campaigns. In model 2, ESM distributed free samples of KOKO Plus^™^ at various venues including at primary healthcare centers and pediatric outpatients clinics in district general hospitals as part of BCC and demand creation activities early in the program. This was stopped after a review of program activities by all stakeholders expressed concerns about the ethics of promoting a commercial product at health facilities. Generic IYCF promotion was also delivered by health extension workers (HEWs) employed by Ghana Health Services (GHS) in the model 2 areas. KOKO Plus^™^ was associated with health services in model 2. The most common source of information and the most common source of KOKO Plus^™^ reported by caregivers in model 2 areas was the health sector in both surveys. The most common source of information and the most common source of KOKO Plus^™^ reported by caregivers in model 1 areas was village-based entrepreneurs in all three surveys. This suggests that health services involvement may not have had the expected positive impact upon coverage.

The main strengths of the work reported here are:

- The surveys assessed both need and coverage and linked them using summary statistics, such as MN and the CR.
- The surveys used standard and validated indicators of need and risk collected using validated instruments
- The surveys used a spatial sample design suited for indicators such as coverage that are likely to exhibit considerable spatial heterogeneity.

The principal weakness of the work reported here is the short duration of the programs being assessed. This may be a particular problem with Delivery Model 2. We cannot rule out that effective coverage would have improved considerably once message and contact coverage reached critical values effecting a socio-behavioral “tipping point” [32] although this appears unlikely given the drop in effective coverage observed between the two survey rounds. It is also unclear whether a small cadre of door-to-door salespersons could achieve similarly high levels of coverage in highly populous urban and peri-urban settings, although the direct marketing model has proved successful in many settings for selling household items, lingerie, sex toys, herbal supplements, and cosmetics. These points should be further investigated.

## Conclusions

Delivery Model 1 performed well in terms of achieved message, contact, and effective coverages. Effective coverage achieved by Delivery Model 2 was low despite increases in message and contact coverages.

Findings from the present work suggest that future programming efforts should use the health extension / microfinance / petty trader approach (Delivery Model 1) in rural settings and consider adapting this approach for use in urban and peri-urban settings. Ongoing BCC and demand creation activities will be essential to the continued success of any such programming.

The work reported here indicates that product availability and brand recognition, while necessary, are not sufficient to deliver effective coverage and impact. The use of social marketing is not a simple alternative for achieving high coverage and impact for health and nutrition interventions. This is likely to be particularly true if the product is one for which there is no pre-existing supply chain, no pre-existing demand (home fortification is a new feeding behavior), and a consumer-base subject to a high and constant churn rate (children enter and leave the consumer-base as they age), and which may offer a low potential for profit. Such products will need skillful and sustained BCC and demand creation work in potential consumers and throughout the supply chain if coverage and impact are to be achieved. Successful use of markets requires hard and exacting work for which there is no magical labor-saving mechanism that can be *easily* harnessed to deliver public goods.

## Supporting Information

S1 Table Strobe Checklist. (DOCX)

## References

[1] WHO/UNICEF. 2003. Global strategy on infant and young child feeding. Geneva, World Health Organization. (http://www.who.int/maternal_child_adolescent/documents/9241562218/en/, accessed on: March 6, 2015).

[2] Effect of breastfeeding on infant and child mortality due to infectious diseases in less developed countries: a pooled analysis. WHO Collaborative Study Team on the Role of Breastfeeding on the Prevention of Infant Mortality. Lancet. 2000;355(9202):451–5. . 10841125

[3] Daelmans B, Mangasaryan N, Martines J, Saadeh R, Casanovas C, Arabi M. Strengthening actions to improve feeding of infants and young children 6 to 23 months of age: summary of a recent World Health Organization/UNICEF technical meeting, Geneva, 6–9 October 2008. Food and nutrition bulletin. 2009;30(2 Suppl):S236–8. . 20496617 10.1177/15648265090302S208

[4] Ghana Statistical Service, 2011. Ghana Multiple Indicator Cluster Survey with an Enhanced Malaria Module and Biomarker, 2011, Final Report. Accra, Ghana. https://goo.gl/UJzQVg. Accessed 20 June 2015.

[5] Ghana Statistical Service, Ghana Health Service, and ICF Macro. Ghana Demographic and Health Survey 2008 [Dataset]. Data Extract from GHIR5A and GHHR5A.SAV. Integrated Demographic and Health Series (IDHS), version 1.0, Minnesota Population Center and ICF International [Distributors]. Accessed from http://idhsdata.org

[6] WHO. 2013. Childhood Stunting: Context, Causes and Consequences. World Health Organization conceptual framework. (http://www.who.int/nutrition/events/2013_ChildhoodStunting_colloquium_14Oct_ConceptualFramework_colour.pdf, accessed on: March 6, 2015).

[7] WHO, IFPRI, UC Davis, FANTA, USAID, UNICEF, Indicators for assessing infant and young child feeding practices: Part 1—Definitions, Geneva, World Health Organization, 2008

[8] WHO, IFPRI, UC Davis, FANTA, USAID, UNICEF, Indicators for assessing infant and young child feeding practices: Part 2—Measurement, Geneva, World Health Organization, 2010

[9] Ghosh S, Tano-Debrah K, Aaron GJ, Otoo G, Strutt N, Bomfeh K, et al Improving complementary feeding in Ghana: reaching the vulnerable through innovative business—the case of KOKO Plus. Annals of the New York Academy of Sciences. 2014;1331:76–89. 10.1111/nyas.12596 . 25514865

[10] FAO. Irrigation in Africa figures—AQUASTAT survey 2005. (available at: http://www.fao.org/nr/water/aquastat/countries_regions/gha/GHA-CP_eng.pdf. Accessed on October 7, 2015).

[11] Bennett S, Radalowicz A, Vella V, Tomkins A. A computer simulation of household sampling schemes for health surveys in developing countries. International journal of epidemiology. 1994;23(6):1282–91. . 7721532 10.1093/ije/23.6.1282

[12] Isserlis L. On the value of a mean as calculated from a sample. Journal of the Royal Statistical Society. 1918;81(1):75–81.

[13] Isaaks E, Srivastava R. An Introduction to Applied Geostatistics, OUP, New York 1989.

[14] Pfeiffer D, Robinson T, Stevenson M, Stevens K, Togers D, ACA. C. Spatial Analysis in Epidemiology, OUP, Oxford 2008.

[15] Anon. 2005. Designing Household Survey Samples: Practical Guidelines, Statistics Division (Studies in Methods, Series F, No.98), Department of Economic and Social Affairs of the United Nations Secretariat, New York, USA.

[16] Piazza T. 2010 Fundamentals of Applied sampling, in Marsen PV, Wright JD (eds). Handbook of Survey Research (2nd Edition), Emerald Group Publishing, Bingley, UK.

[17] Lauritsen JM, Bruus M, EpiData Entry v3.1: A comprehensive tool for validated entry and documentation of data, The EpiData Association, Odense Denmark, 2004.

[18] Myatt M, Guevarra E, Fieschi L, Norris A, Guerrero S, Schofield L, et al. Semi-Quantitative Evaluation of Access and Coverage (SQUEAC) / Simplified Lot Quality Assurance Evaluation of Access and Coverage (SLEAC) Technical Reference, Food and Nutritional technical Assistance III Project (FANTA-III), FHI 360 / FANTA, Washington, DC, October 2012.

[19] Alkire S, Santos ME. Measuring Acute Poverty in the Developing World: Robustness and Scope of the Multidimensional Poverty Index. World Development. 2014;59(0):251–74. 10.1016/j.worlddev.2014.01.026.

[20] Kennedy G, Ballard T, Dop M. Guidelines for Measuring Household and Individual Dietary Diversity, Nutrition and Consumer Protection Division, Food and Agriculture Organization of the United Nations, Rome, 2010 (http://www.fao.org/3/a-i1983e.pdf, accessed on March 24, 2015).

[21] Arimond M, Ruel M. Generating Indicators of Appropriate Feeding of Children 6 through 23 months from the KPC 2000+, Washington DC, FANTA / AED, 2003 (http://www.micronutrient.org/nutritiontoolkit/ModuleFolders/3.Indicators%5CDietary%5CResources%5CFANTA_Complementary_feeding_indicators.pdf, accessed on March 24, 2015).

[22] Guevarra E, Siling K, Chiwile F, Mutunga M, Senesie J, Beckley W, et al IYCF assessment with small-sample surveys: A proposal for a simplified and structured approach, Field Exchange, 2014;47:60–63.

[23] Tanahashi T. Health service coverage and its evaluation. Bulletin of the World Health Organization. 1978;56(2):295–303. 96953 PMC2395571

[24] Cameron A, Gelbach J, Miller D. Bootstrap-based improvements for inference with clustered errors. Review of Economics and Statistics. 2008;90:414–427.

[25] Baker J. Reducing Bias and Inefficiency in the Selection Algorithm, in Grefenstette JJ (editor), Proceedings of the Second International Conference on Genetic Algorithms and their Application, Lawrence Erlbaum Associates, Hillsdale, New Jersey, USA, 1987.

[26] Efron B, Tibshirani R. An Introduction to the Bootstrap, Chapman & Hall / CRC, Boca Raton, USA, 1993.

[27] Geisser S. The predictive sample reuse method with applications, J. Amer. Statist. Assoc., 1975;70:320–328.

[28] Shepardd A. Two-dimensional interpolation function for irregularly spaced data, Proceedings of the 1968 ACM National Conference (517–524), 1986.

[29] Guevarra E, Guerrero S, Myatt M, Considerations regarding coverage standards for selective feeding programmes, Field Exchange, 2013;46:19–20.

[30] Dekimpe MG, Hanssens DM. The Persistence of Marketing Effects on Sales. Marketing Science. 1995;14(1):1–21. 10.1287/mksc.14.1.1

[31] Suchdev PS, Shah A, Jefferds ME, Eleveld A, Patel M, Stein AD, et al Sustainability of market-based community distribution of Sprinkles in western Kenya. Maternal & child nutrition. 2013;9 Suppl 1:78–88. 10.1111/j.1740-8709.2012.00450.x . 23167586 PMC6860854

[32] Morton G. Metropolitan Segregation. 1957 Scientific American 197:33–47.

